# P-1813. The natural history of respiratory viral antibodies during low circulation of respiratory viruses during the COVID-19 pandemic

**DOI:** 10.1093/ofid/ofaf695.1982

**Published:** 2026-01-11

**Authors:** Sara R Kim, Linda M Sircy, Yun Lim, Khaleel Yahya, Terry L Stevens-Ayers, Nina Ozbek, Rachel Blazevic, Larry Mose, Louise E Kimball, Michael J Boeckh, Alpana Waghmare

**Affiliations:** Seattle Children's Hospital, Seattle, WA; Fred Hutchinson Cancer Center, Seattle, Washington; Fred Hutch Cancer Center, Seattle, Washington; Fred Hutchinson Cancer Center, Seattle, Washington; Fred Hutchinson Cancer Center, Seattle, Washington; Fred Hutchinson Cancer Center, Seattle, Washington; Fred Hutchinson Cancer Center, Seattle, Washington; Fred Hutchinson Cancer Center, Seattle, Washington; Fred Hutchinson Cancer Center, Seattle, Washington; Fred Hutchinson Cancer Center, Seattle, Washington; Seattle Children's Hospital/Fred Hutchinson Cancer Center, Seattle, Washington

## Abstract

**Background:**

Social distancing measures during the COVID-19 pandemic disrupted the circulation of most seasonal respiratory viruses (RV), providing an unprecedented opportunity to assess the waning of RV antibodies. Using a pan-viral immunosurvey, we assessed RV immunity during a prolonged time of low RV circulation at a population level.

**Figure 1.**
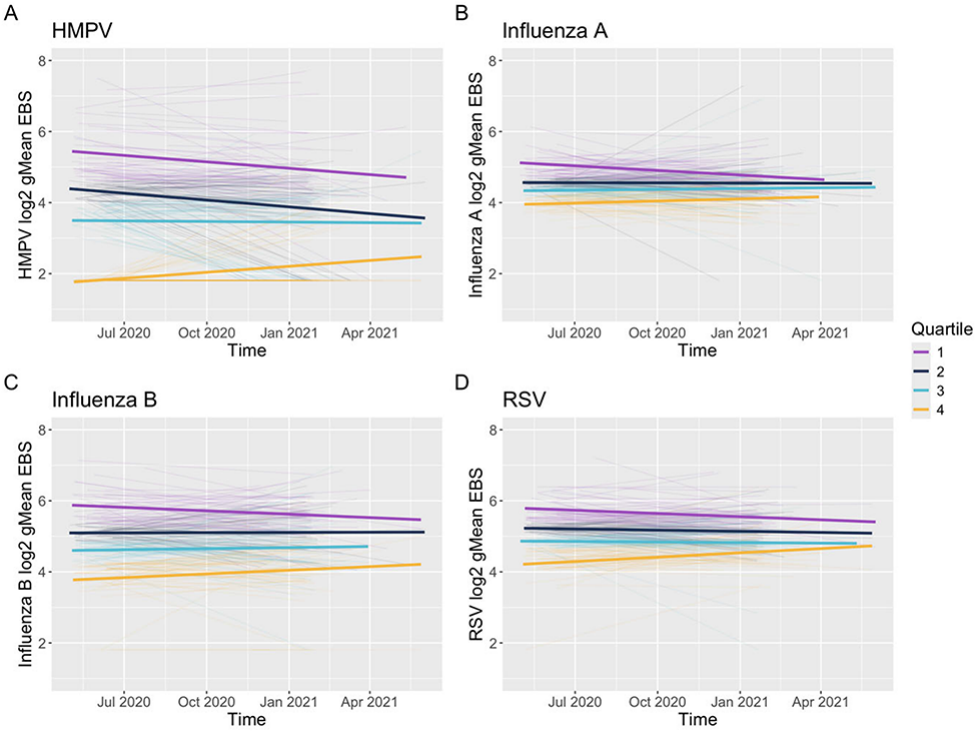
The longitudinal waning of respiratory viral antibodies during the COVID-19 pandemic. Linear mixed effect model for: (A) human metapneumovirus [HMPV], (B) influenza A, (C) influenza B , and (D) respiratory syncytial virus [RSV]. Each thin line represents a patient with geometric mean (gMean) epitope binding signal (EBS) at enrollment and end of study. Quartile 1 (purple) represents the patients with the highest gMean EBS at enrollment and quartile 4 (orange) represents the patients with the lowest gMean EBS. The thick lines represented predicted spline lines for each quartile based on the population.

**Figure 2.**
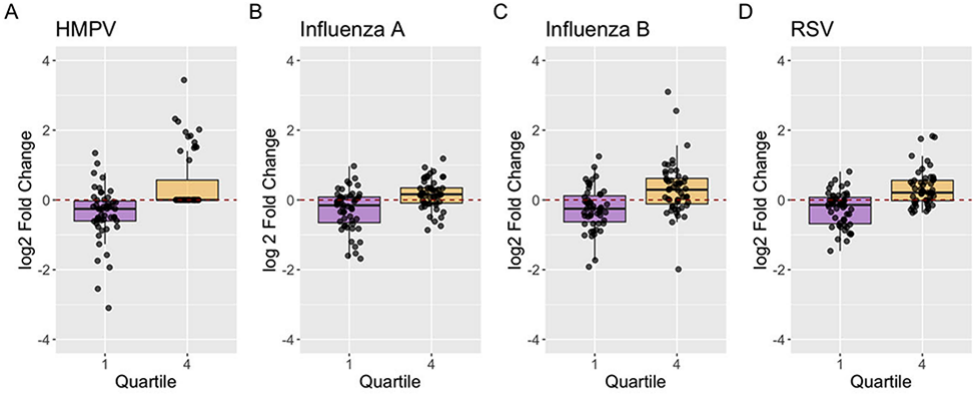
The log2-transformed fold changes in geometric mean epitope binding signal (EBS), a surrogate of antibody titer, for four different respiratory viruses. Boxplots show the distribution of log_2_-transformed fold changes in antibody levels for each virus: (A) Human metapneumovirus (HMPV), (B) Influenza A, (C) Influenza B, and (D) Respiratory Syncytial Virus (RSV). Individual points represent participant-level values. A log_2_ fold change of 0 indicates no change in antibody level; values below 0 indicate waning, while values above 0 indicate an increase. Comparisons between quartile 1 and quartile 4 were assessed using the Wilcoxon rank-sum test. All comparisons had a p<0.0001.

**Methods:**

VirScan, an immunosurvey that can identify antibodies against viruses with human tropism, was performed on samples from a cohort of patients in a SARS-CoV-2 surveillance study from May 2020 to June 2021. The median study period was 229 days. We selected 4 RVs (human metapneumovirus [HMPV], influenza A and B, and respiratory syncytial virus. The enrollment samples were ranked into quartiles (Q1-Q4) based on the geometric mean (gMean) epitope binding signal (EBS), a VirScan surrogate for an antibody titer. Q1 represented the highest gMean EBS, likely due to recent RV exposure. We used linear mixed effect (LME) models to model longitudinal changes in gMean EBS for each RV and calculated fold change (ratio of gMean EBS at end of study to enrollment). Fold change values were log₂-transformed for visualization and statistical analysis.

**Results:**

A total of 204 immunocompetent adults were included [median age 43.5 (IQR: 32.3 – 54.8)]. LME models showed a consistent decline in gMean EBS over time for Q1 compared to Q4, which demonstrated a modest increase or stability in antibody levels (Figure 1A-D). This trend was most pronounced for HMPV, which also exhibited greater variability in response compared to other RVs (Figure 1A). The log_2_-transformed fold change in gMean EBS between timepoints was compared for each RV between Q1 and Q4. Q1 fold changes were significantly greater than Q4 for all RVs, indicating greater waning (Fig. 2A-D, P< 0.0001).

**Conclusion:**

We demonstrate that VirScan can assess population level changes in antibody repertoires. Our results show waning humoral immunity across 4 RV during a period of low circulation in patients with the highest EBS (antibody titers) at enrollment. We were able to assess antibody levels for multiple viruses using a single assay, thus allowing for broad immune profiling compared to traditional methods of assessing antibody kinetics. Future studies will utilize VirScan to assess changes at an epitope level.

**Disclosures:**

Michael J. Boeckh, MD PhD, Allovir: Advisor/Consultant|Ansun Biopharma: Grant/Research Support|AstraZeneka: Advisor/Consultant|AstraZeneka: Grant/Research Support|GSK: Grant/Research Support|Merck: Advisor/Consultant|Merck: Grant/Research Support|Moderna: Advisor/Consultant|Moderna: Grant/Research Support|Symbio: Advisor/Consultant|Vir Biotechnology: Grant/Research Support Alpana Waghmare, MD, Ansun Biopharma: Clinical trial site|AstraZeneca: Advisor/Consultant|GSK: Advisor/Consultant|GSK: Grant/Research Support|Merck: Advisor/Consultant|Merck: Grant/Research Support|Pfizer: Clinical trial site|Shionogi: Clinical trial site|Vir Biotechnology: Advisor/Consultant

